# Genomic profiling of Nipah virus using NGS driven RNA-Seq expression data

**DOI:** 10.6026/97320630015853

**Published:** 2019-12-31

**Authors:** Md. Zakiul Hassan, Md. Shakil Ahmed, Md. Marufuzzaman Khan, Mohammad Ahsan Uddin, Fahmida Chowdhury, Md Kamruzzaman

**Affiliations:** 1 Infectious Diseases Division, International Centre for Diarrheal Disease Research, Bangladesh, Dhaka, Bangladesh; 2 Department of Public Health, The University of Tennessee, Knoxville, Tennessee, USA; 3 Department of Statistics, University of Dhaka, Dhaka, Bangladesh; 4 Institute of Bangladesh Studies, University of Rajshahi, Rajshahi, Bangladesh

**Keywords:** Nipah virus, NGS RNA-Seq, limma, Phylogenetic gene tree, Protein-protein interaction network

## Abstract

Nipah virus (NiV) is an ssRNA, enveloped paramyxovirus in the genus Henipaveridae with a case fatality rate >70%. We analyzed the NGS RNA-Seq gene expression data of NiV to detect differentially expressed genes (DEGs) using the statistical R package limma. We used the Cytoscape, Ensembl, and STRING tools to construct the gene-gene interaction tree, phylogenetic gene tree and protein-protein interaction networks towards functional annotation. We identified 2707 DEGs (p-value <0.05) among 54359 NiV genes. The top-up and down-regulated DEGs were EPST1, MX1, IFIT3, RSAD2, OAS1, OASL, CMPK2 and SLFN13, SPAC977.17 using log2FC criteria with optimum threshold 1.0. The top 20 up-regulated gene-gene interaction trees showed no significant association between Nipah and Tularemia virus. Similarly, the top 20 down-regulated genes of neither Ebola nor Tularemia virus showed an association with the Nipah virus. Hence, we document the top-up and down-regulated DEGs for further consideration as biomarkers and candidates for vaccine or drug design against Nipah virus to combat infection.

## Background

Nipah virus (NiV) is a stage III zoonotic pathogen from the family of Paramyxoviridae and a new genus from the Henipavirus [1]. Nipah virus was first discovered in a large encephalitis outbreak in Malaysia in 1998 [2-4]. Nipah virus outbreak has been recognized nearly every year in Bangladesh since 2001 and occasionally in neighboring India [5-9]. With the capacity of person-to-person transmission, high case fatality rate (>70%) and no availability of treatment or vaccine, the World Health Organization included the Nipah virus among the 7 Blueprint list of priority diseases and effort for Nipah vaccine development is underway [10-12]. Genes are strongly involved in NiV infection in interferon response in endothelial cells. The chemokine CXCL10 (interferon-induced protein 10, IP-10) gene was identified among the top 10 up- regulated genes. The cellular functionality of CXCL10 is a generation of inflammatory immune response and neurotoxicity [13]. Arankalle, V. A et. al. performed the NiV whole-genome sequencing (18,252 nucleotides) from the lung tissue samples [14]. Detection of DEGs is an important branch of transcriptomics research in bioinformatics. RNA-sequencing (RNA-seq) is the modern Next Generation Sequencing (NGS) technology for genomic profiling of any bacteria, virus or pathogens and other causes of diseases. Identification of DEGs or transcripts associated with the specific trait of interest from the high dimension of transcriptomic data based on NGS RNA-Seq gene expression technique. Previously microarray technology had been used by biological and biomedical researchers for discovering the candidate genes and differentially expressed markers between two or more groups of interest. Additionally, this approach includes the identification of disease biomarkers that may be important in the diagnosis of the different types and subtypes of diseases, with several implications in terms of prognosis and therapy [15]. This sequence-based technology has created significant scope for studying the transcriptome and enabling a wide range of novel applications, including detection of alternative splicing isoforms [16-19], detecting novel genes, gene promoters, isoforms, and allele-specific expression [20]. RNA-seq uses NGS technology to sequence cDNA that has been derived from an RNA sample, and hence generates millions of short reads [21]. One important objective for RNA-seq is to identify DEGs under different conditions. Researchers typically target for differential expression analysis called "count matrix", where each row represents the gene, each column represents the sample, and each cell indicates the number of reads mapped to the gene in the sample [22]. A basic research problem in many RNA-seq analyses is the discovery of DEGs between different sample groups (e.g. healthy and disease). RNA-seq analysis has some benefits over microarrays for DE analysis including wide dynamic range and a lower background level, and the chance to detect and quantify the expression of previously unknown transcripts [23]. Identification of differentially expressed genes from the large scale NGS RNA-Seq data and functional annotation of the Nipah virus were the key objectives of this study.

## Methodology

### NGS RNA-Seq Microarray Gene Expression Dataset: 

We used Microarray gene expression of RNA-Seq data for molecular investigation of NiV infection. We collected the complete genome of selected pathogen from the National Centre for Biotechnology Information (NCBI). To analyze data we considered 7 (seven) different datasets with accession numbers in the GEO (Gene Expression Omnibus) database are as follows GSE32902, GSE23986, GSE93861, GSE18064, GSE12108, GSE69980, GSE89915 [13,[24-29].

Download links for data are as follows:

[1] NCBI Gene Expression Omnibus (https://www.ncbi.nlm.nih.gov/geo/)

[2] Nipah Virus (https://www.ncbi.nlm.nih.gov/geo/query/acc.cgi?acc=GSE32902)

[3] Dengue virus type-3 (https://www.ncbi.nlm.nih.gov/geo/query/acc.cgi?acc=GSE23986)

[4] Ebola virus (https://www.ncbi.nlm.nih.gov/geo/query/acc.cgi?acc=GSE93861)

[5] Rift Valley Fever virus (https://www.ncbi.nlm.nih.gov/geo/query/acc.cgi?acc=GSE18064)

[6] Tularemia (https://www.ncbi.nlm.nih.gov/geo/query/acc.cgi?acc=GSE12108)

[7] Chikungunya (https://www.ncbi.nlm.nih.gov/geo/query/acc.cgi?acc=GSE69980)

[8] Zika virus (https://www.ncbi.nlm.nih.gov/geo/query/acc.cgi?acc=GSE89915)

### Statistical Analysis:

**Figure 1:**
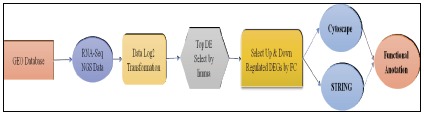
The schematic diagram of the work flow of the study of genomic profiling of Nipah virus

We performed the log2 transformation to analyze the RNA-Seq NGS gene expression data. The log transformation is the robust method for scaling and controlling the outliers of the dataset by uniform pattern.Linear model for microarray analysis (limma) is the efficient statistical tool integrated in R package for identification of DEGs from large scale expression data [30]. The log2FC (Fold Change) = log2 {mean (Infected)/mean (Normal)} method was used for selection of up and down-regulated DEGs [31]. The p-value was calculated using a t-test statistic from the linear model. The Benjamini and Hochberg (False discovery rate) was used for calculation of the adjusted p-value. All the statistical analysis was implemented using R statistical programming language software. For functional annotation and biological network of differentially expressed genes were analyzed using STRING, Ensembl and Cytoscape bioinformatics tools respectively.(Figure 1)shows the work flow of the manuscript.

## Results and Discussion:

### Characteristics of Nipah virus dataset: 

The row represents the gene and the column represents the RNA samples in the NGS RNA-Seq data matrix of Nipah virus (GEO ID: GSE32902). In this dataset four samples (two uninfected HUVEC and two NiV-infected HUVEC) and 54359 genes were included.

### Differentially Expressed Genes (DEGs) Detection:

We used t-test statistic to calculate the p-values for each gene (54359) of Nipah virus dataset. We used a cut of point less than 0.05 at a 5% level of significance to select the DEGs. We identified 2707 DEGs with this cut of value. Similarly, for Chikungunya virus 1544 DEGs out of 45220 genes were identified using a p value<0.05 cut of point. We identified 1200 DEGs (p value<0.05) of 33297 genes of the Dengue type III virus. The Ebola Virus RNA-Seq dataset included 41000 genes and of these 20211 genes were detected as DEGs using a p value<0.05 in t-test statistic. With the same p-value cut of point (<0.05), we identified 14470 DEGs of 54675 genes for Tularemia (Francisella tularensis), 6666 DEGs of 45101 genes for Rift valley fever virus and, 688 DEGs of 43376 genes for Zika virus.

### Up and Down Regulated DEGs Detection:

We used the log2 fold change or a cutoff at 0.5 for down and 1/2 for up-regulated genes and selected genes under/above thresholds. A log2FC-value close to 0.5 corresponds to equally expressed (EE) genes, while down- and up-regulated genes have log2FC-values close to 0 and 1 correspondingly [20]. In this study, log2FC cut of value 1 was used for up- and down-regulated DEGs selection for all viruses except dengue type III (log2FC cut of 0.5). With the log2FC cut of thresholds, 834 up- (log2FC>1.0) and 1873 down-regulated (log2FC≤1.0) DEGs in Nipah virus, 743 up- and 801 down-regulated DEGs in Chikungunya, 8 up- (log2FC>0.5) and 1192 down-regulated (log2FC≤0.5) DEGs in Dengue type III, 3271 up- and 16940 down-regulated DEGs in Ebola virus, 251 up- and 14219 down-regulated DEGs in Tularemia, 1204 up- and 5462 down-regulated DEGs in Rift valley fever virus, and 338 up- and 350 down-regulated DEGs in Zika virus.

### Gene-Gene Interaction Network:

**Figure 2:**
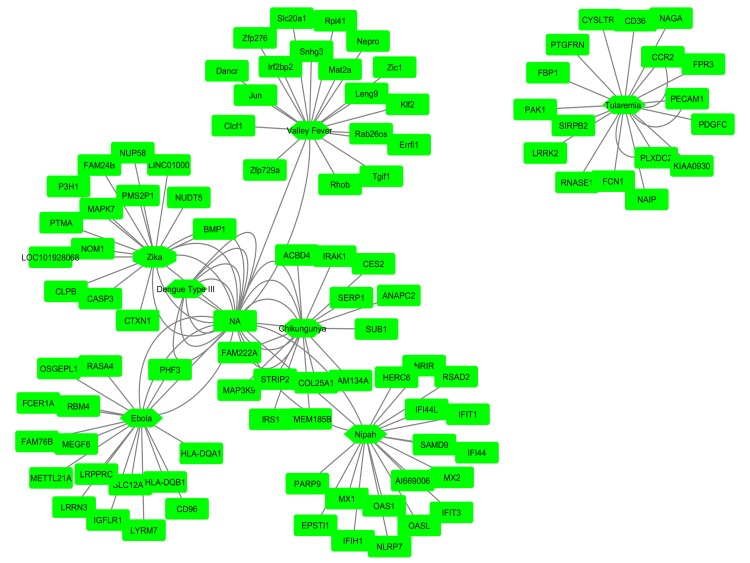
Association among zoonotic viruses including Nipah virus using gene-gene interaction network tree for top 20 up regulated DE genes

**Figure 3:**
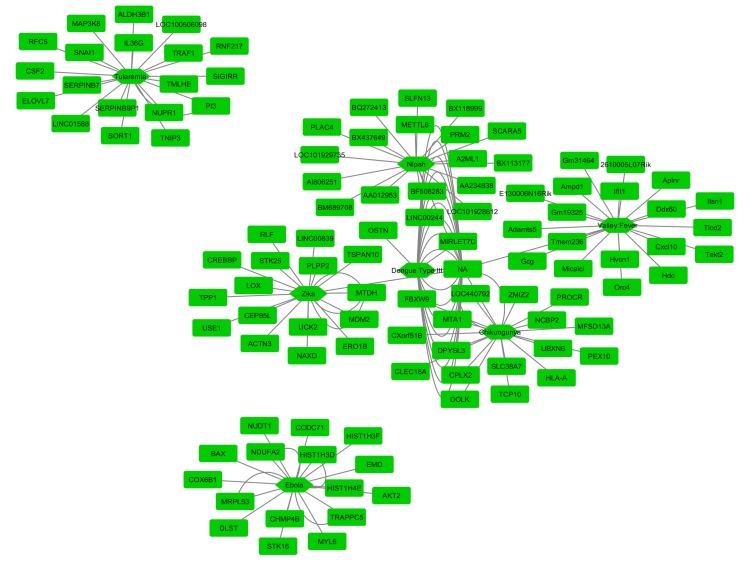
Association among zoonotic viruses including Nipah virus using gene-gene interaction network tree for top 20 down regulated DE genes

We used Cytoscape (https://cytoscape.org/) bioinformatics tool to construct a gene-gene interaction network. Based on the calculated p-value, we selected top 20 up- and down-regulated genes (p-value <0.000481 and log2FC>1) for 7 viruses (Nipah, Chikungunya, Dengue type III, Ebola, Tularemia, Valley fever, Zika) ranked according to increasing order for each gene. The gene-gene interaction tree of the top 20 up-regulated DEGs among the 7 viruses showed that the Tularemia virus had no association with the other 6 viruses including the Nipah virus and, it represents a separate gene tree. The other five viruses, however, showed that a significant association with the Nipah virus and unknown gene symbol (NA; not available) and a strong network was seen among these viruses (Figure 2). From the gene-gene interaction tree of the top 20 down-regulated DEGs, we found that the Ebola and Tularemia virus showed no association with the other five viruses and showed a separate pattern. Nipah virus was strongly associated with Chikungunya, Dengue type III, Valley fever, and Zika virus. The unknown gene symbol (NA) showed a strong association with Nipah, Chikungunya, Dengue type III, Rift Valley fever, and Zika viruses (Figure 3).

### Phylogenetic Gene Tree for top DE genes of Nipah Virus:

**Figure 4:**
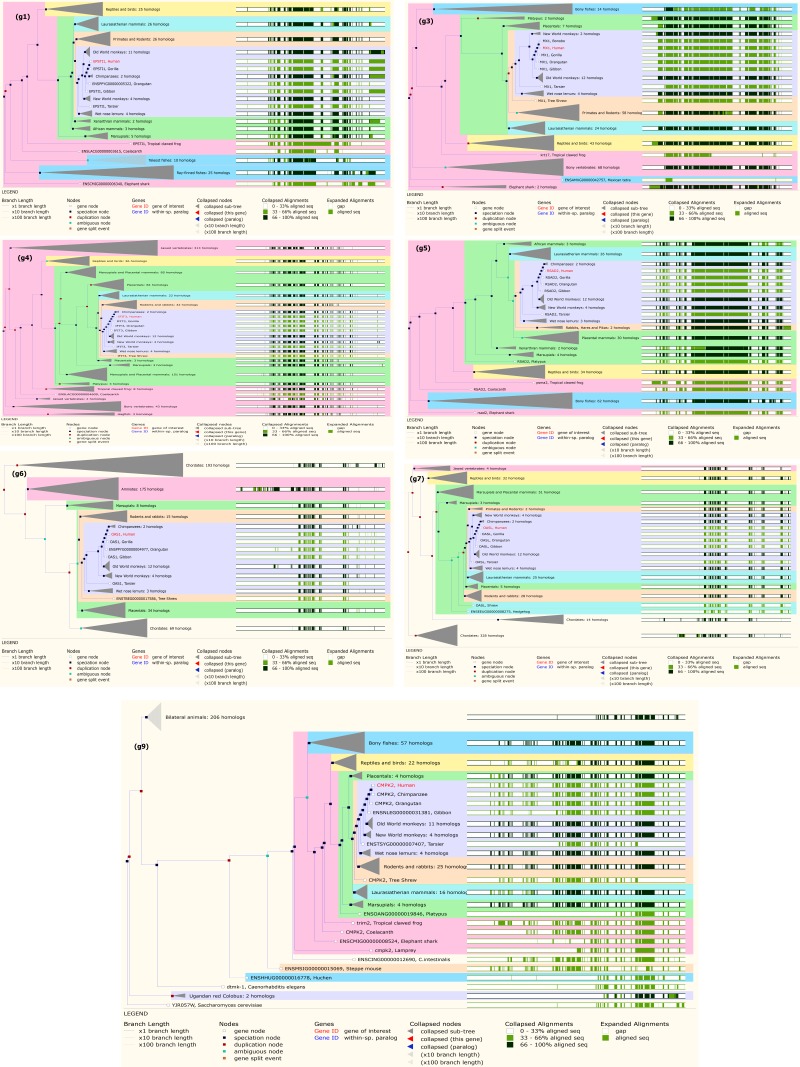
Gene tree for genomic classification of top up regulated seven functional DE genes: (g1) EPST1; (g3) MX1; (g4) IFIT3; (g5) RSAD2; (g6) OAS1; (g7) OASL; (g9) CMPK2

We selected the top 10 up-regulated genes using ranking p-value. Of 10, 7 were functional: EPST1 (g1), MX1 (g3), IFIT3 (g4), RSAD2 (g5), OAS1 (g6), OASL (g7), CMPK2 (g9) and 3 were non-functional genes (Table 1).We used the Ensembl (https://asia.ensembl.org/index.html) online bioinformatics tool for the construction of the phylogenetic gene tree of top up-regulated functional DEGs. We found that EPST1, Human gene were similar cluster group with EPST1, Gorilla (Figure 4 (g1)); MX1, Human gene highly similar cluster group with MX1, Gorilla (Figure 4 (g3) ); IFIT3, Human gene highly similar cluster group with Chimpanzees: 2 homologs (Figure 4 (g4)); RSAD2, Human gene highly similar cluster group with Chimpanzees: 2 homologs (Figure 4 (g5)); OAS1, Human gene highly similar cluster group with Chimpanzees: 2 homologs (Figure 4 (g6)); OASL, Human gene highly similar cluster group with Chimpanzees: 2 homologs (Figure 4 (g7)); CMPK2, Human gene highly similar cluster group with CMPK2, Chimpanzee (Figure 4(g9) ) from the phylogenetic gene tree (Figure 4). The functional down-regulated DEGs SLFN13, Human highly similar cluster group with Chimpanzees: 2 homologs from the phylogenetic gene tree (Table 2 and Figure 6(c)).

### Protein-protein Interaction Network:

**Figure 5:**
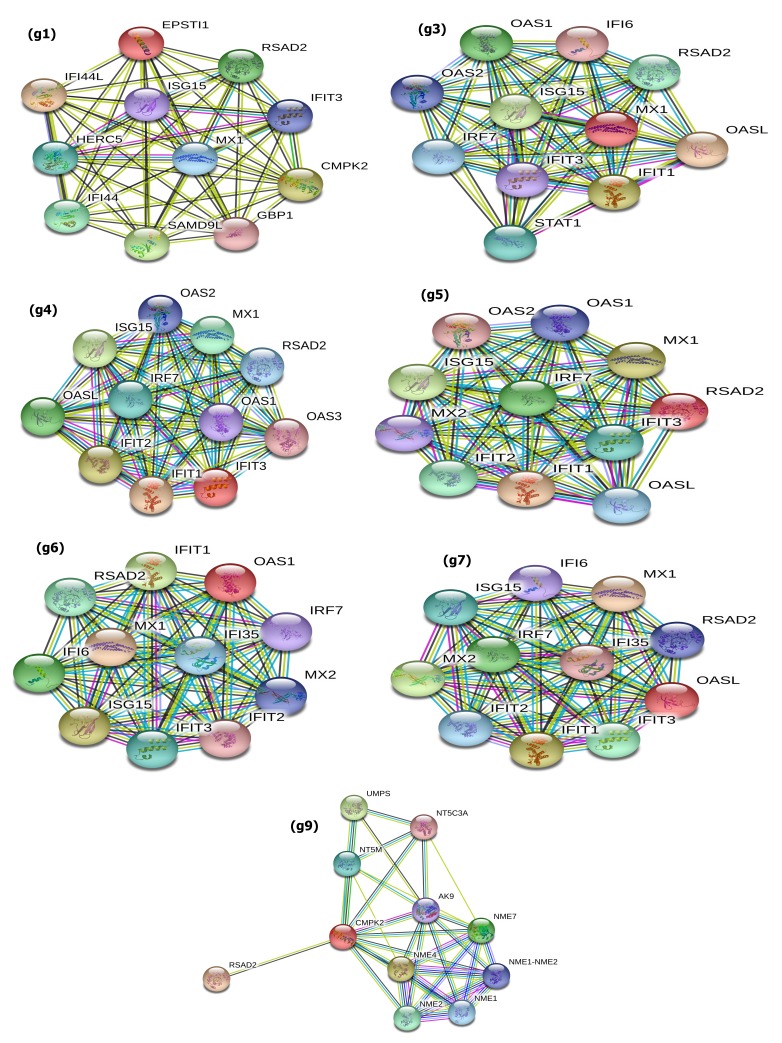
Protein-protein interaction network for top up regulated seven functional DE genes (red color node is the target gene node): (g1) EPST1; (g3) MX1; (g4) IFIT3; (g5) RSAD2; (g6) OAS1; (g7) OASL; (g9) CMPK2

**Figure 6:**
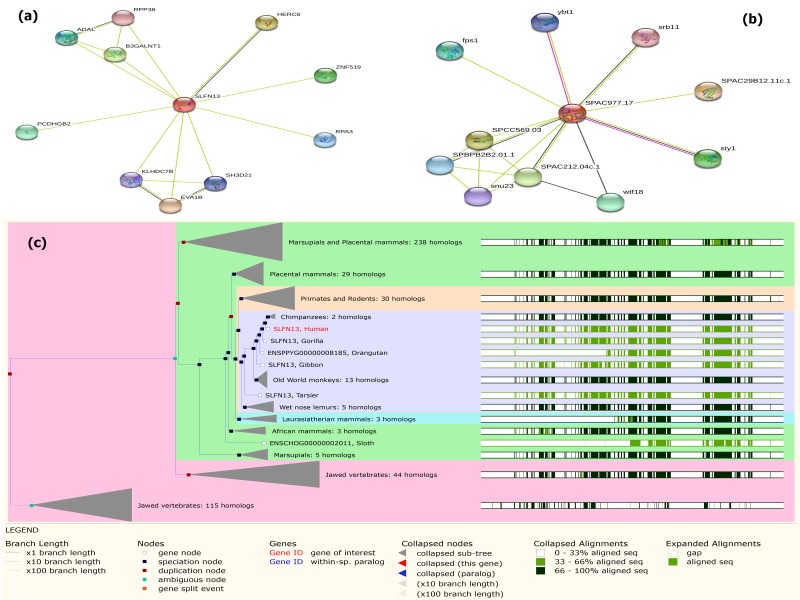
Protein-protein interaction network and phylogenetic gene tree for top down regulated two functional DE genes. (a) PPI network of SLFN13 (b) PPI network of SPAC977.17 (C) phylogenic gene tree of SLFN13

We used the STRING (https://string-db.org/) online bioinformatics tool for protein-protein interaction (PPI) networks functional enrichment analysis. Top up-regulated seven functional EPST1 (g1), MX1 (g3), IFIT3 (g4), RSAD2 (g5), OAS1 (g6), OASL (g7), CMPK2 (g9) DEGs of Nipah virus were highly interacting proteins by score with IFI44L, OASL, IFIT1, IFIT1, MX1, MX1, and RSAD2 respectively. All the PPI network enrichment was statistically significant (p value<0.05) at a 5% level of significance (Figure 5).All top interacting genes were interferon (INF) induced proteins that exist in the antiviral functional pathway (Table 3). Previous analysis by Nicole B et al. who performed the initial genomic profile of virus-induced innate immune response in Pteropus vampyrus bats, a known reservoir of the Nipah virus. The study found that henipavirus IFN antagonist mechanisms are likely active in bat cells [32-33]. Similarly the top down-regulated functional DEGs SLFN13 and SPAC977.17 were best interaction with EVA1B (0.474; p value=0.0613) and SPAC29B12.11c.1 (0.882; p value=0.0389) proteins according to score and p-value (<0.05) (Table 3;Figure 6(a) and (b) ).

## Conclusions:

We used the statistical R package limma to analyze the NGS RNA-seq data to detect DEGs (biomarker) of the Nipah virus for application in combat and care of the disease. We identified 2707 DEGs (p-value <0.05) among the 54359 genes of the virus. We report 834 up-regulated and 1873 down-regulated DEGs estimated by the log2FC approach at threshold value 1.0. This data will help in the selection of biomarkers and vaccine targets against the virus.

## Figures and Tables

**Table 1:** Top 10 up regulated genes of Nipah virus

ID	adj. P Value	P Value	t	B	Log FC	Gene symbol	Function Status
426044	0.884	7.55E-05	22.7	-0.831	3.82	EPSTI1	Functional
82039	0.884	0.0001	20.9	-0.853	4.52	AI669006	Non-functional
404052	0.884	0.000103	20.8	-0.854	3.8	MX1	Functional
190107	0.884	0.000135	19.2	-0.879	3.43	IFIT3	Functional
255017	0.884	0.000142	18.9	-0.884	3.52	RSAD2	Functional
304097	0.884	0.000143	18.8	-0.885	3.11	OAS1	Functional
60082	0.884	0.000155	18.4	-0.893	3.44	OASL	Functional
141045	0.884	0.000177	17.7	-0.907	3.45	Unknown	Unknown
442005	0.884	0.000186	17.4	-0.913	2.95	CMPK2	Functional
123024	0.884	0.000189	17.4	-0.915	4.52	Unknown	Unknown

**Table 2:** Top 10 down regulated genes of Nipah virus

ID	adj. P Value	P Value	t	B	logFC	Gene symbol	Function Status
213081	0.884	0.000223	-16.5	-0.934	-4.27	Unknown	Unknown
444022	0.884	0.000234	-16.3	-0.94	-2.73	LINC00244	Non-functional
290080	0.884	0.000294	-15.2	-0.972	-2.54	AI806251	Non-functional
493014	0.884	0.00035	-14.5	-0.998	-3.1	LOC101928812	Non-functional
46044	0.884	0.000361	-14.3	-1.003	-5.32	SLFN13	Functional
383031	0.884	0.000429	-13.6	-1.033	-2.4	LOC101929735/TRDV1	Non-functional
74032	0.884	0.000529	-12.8	-1.071	-2.51	BQ272413	Non-functional
475050	0.884	0.000592	-12.4	-1.094	-2.07	BM689708	Non-functional
333045	0.884	0.000603	-12.3	-1.098	-2.62	PLAC4	Non-functional
106012	0.884	0.000646	-12	-1.112	-2.13	AQP7P1	Functional

**Table 3:** Most predicted partners for top functional up- and down-regulated functional DEGs of Nipah virus

Gene No.	Target genes	Most predicted partners			P value
		Gene symbol	Function	Score	
(g1)	EPSTI1 (Epithelial stromal interaction 1; 410 aa)	IFI44L (Interferon-induced protein 44-like; 452 aa)	Exhibits a low antiviral activity against hepatitis C virus	0.736	<1.0e-16
(g3)	MX1 (Interferon-induced GTP-binding protein Mx1; 662 aa)	OASL(2/-5/-oligoadenylate synthase-like protein)	Does not have 2/-5/-OAS activity, but can bind double-stranded RNA	0.999	< 1.0e-16
(g4)	IFIT3 (Interferon-induced protein with tetratricopeptide repeats 3; 490 aa)	IFIT1 (Interferon-induced protein with tetratricopeptide repeats 1)	Interferon-induced antiviral RNA-binding protein	0.999	< 1.0e-16
(g5)	RSAD2 (Radical S-adenosyl methionine protein 2; 361 aa)	IFIT1 (Interferon-induced protein with tetratricopeptide repeats 1)	Interferon-induced antiviral RNA-binding protein	0.997	< 1.0e-16
(g6)	OAS1 (OASL(2/-5/-oligoadenylate synthase 1; 414 aa)	MX1 (Interferon-induced GTP-binding protein Mx1)	Interferon-induced dynamin-like GTPase with antiviral activity	0.997	< 1.0e-16
(g7)	OASL (2/-5/-oligoadenylate synthase-like protein)	MX1 (Interferon-induced GTP-binding protein Mx1)	Interferon-induced dynamin-like GTPase with antiviral activity	0.999	< 1.0e-16
(g9)	CMPK2 (UMP-CMP kinase 2; 449 aa)	RSAD2 (Radical S-adenosyl methionine domain-containing protein 2)	Interferon-induced inron-sulfur (4FE-4S) cluster-binding activity	0.967	2.62E-08
Top down-regulated functional DEGs of Nipah virus					
(g5)	SLFN13 (Schlafen family member 13; 897 aa)	EVA1B(Eva-1 homolog B; 165 aa)	Belongs to the EVA1 family	0.474	0.0613
(g10)	SPAC977.17 (Uncharacterized membrane protein C977.17; 598 aa)	SPAC29B12.11c.1 (UPF0664 stress-induced protein C29B12.11c)	Human WW domain binding protein-2 ortholog)	0.882	0.0389
